# Comparison of the Time-Dependent Changes in Immediate Early Gene Labeling and Spine Density Following Abstinence From Contingent or Non-contingent Chocolate Pellet Delivery

**DOI:** 10.3389/fnbeh.2018.00144

**Published:** 2018-07-16

**Authors:** Erin W. Noye Tuplin, Savannah H. M. Lightfoot, Matthew R. Holahan

**Affiliations:** Department of Neuroscience, Carleton University, Ottawa, ON, Canada

**Keywords:** FosB, c-Fos, craving, dendritic spines, food reward, operant conditioning, classical conditioning

## Abstract

**Rationale**: Incubation of craving is a phenomenon whereby responding for cues associated with a reward increases over extended periods of abstinence. Both contingent and non-contingent behavioral designs have been used to study the incubation of craving phenomenon with differing results. The present study directly compares behavioral and neural changes following contingent or non-contingent administration of chocolate flavored pellets.

**Objective**: The current study examined whether an incubation of craving response would be observed at the behavioral and neural levels following delays of abstinence from chocolate pellets in a contingent or non-contingent reinforcement design.

**Methods**: Rats were trained for 10 days to bar press for chocolate pellets (contingent) or received chocolate pellets in a non-contingent design (classical conditioning). Groups were then subjected to abstinence from the reward for 24 h, 7, 14 or 28 days at which point they were tested for responding for reward associated cues. Following the test, brains from all rats were processed and assessed for c-Fos and FosB labeling as well as dendritic spine density in the nucleus accumbens (NAc).

**Results**: Behavioral measures during the test (lever presses, food hopper entries and locomotor activity) revealed similar behavioral outcomes across all delays indicating the lack of an incubation of craving response on both the contingent and non-contingent designs. Overall, labeling of c-Fos in the NAc was lower for the non-contingent group compared to the operant-trained and food restricted control. Compared to the operant-trained and non-trained control groups, a significantly reduced FosB labeling was noted in the NAc of the classically conditioned groups across all abstinence periods. Spine density in the NAc was elevated in both the classically and operant conditioned compared to the food-restricted, non-trained controls.

**Conclusions**: Chocolate pellet reward did not result in incubation of craving but did produce behavioral learning that was associated with increased spine density. This suggests that chocolate pellet administration results in long-term structural and functional changes that are present for at least 28 days following abstinence. Contingent and non-contingent administration resulted in differential immediate early gene labeling in the NAc, but the functional significance of this has yet to be elucidated.

## Introduction

Relapse following a period of withdrawal from an addictive substance is often associated with craving, a poorly understood phenomenon that precedes drug-seeking behaviors (Tiffany and Wray, 2012; Sayette, 2016). Craving can be triggered by cues previously associated with drug-taking (Veilleux and Skinner, 2015). These cues include the drug itself as well as sights, sounds, odors, and locations associated with the drug-taking experience (O’Brien et al., 1992; Harkness et al., 2010). Exposure to these drug-related cues may evoke physiological changes that result in craving and contribute to subsequent drug seeking and taking behaviors (Veilleux and Skinner, 2015; Sayette, 2016). Craving may continue even after physiological withdrawal symptoms have subsided (Grimm et al., 2002, 2016; Lu et al., 2004; Pickens et al., 2011). Animal models of drug craving suggest that the onset of craving may be delayed, and even enhanced, following a period of abstinence from a substance. These time-dependent changes in craving following a period of abstinence are termed “incubation of craving” (Grimm et al., 2001; Lu et al., 2004; Grimm et al., 2011).

The majority of studies investigating the incubation of craving focus on self-administration (contingent) of a rewarding substance in the presence of discrete environmental cues. In self-administration models, craving is defined as increased responding on the correct lever where depressing the lever results in the discrete cue(s) previously paired with the reward. Studies involving the self-administration of cocaine have shown that following a period of abstinence, rats have increased cue-induced responding on day 30 compared to day 1 of withdrawal (Grimm et al., 2001, 2002; Lu et al., 2004; Calu et al., 2007; Halbout et al., 2014). An incubation effect has also been observed following self-administration of methamphetamine (Shepard et al., 2004; Li et al., 2015a,b) nicotine (Abdolahi et al., 2010; Funk et al., 2016) heroin (Shalev et al., 2001) and alcohol (Bienkowski et al., 2004).

Incubation of craving for reward has also been studied using non-contingent models of substance administration, but when non-contingent models of administration are utilized, the effect is less consistent. Diehl et al. (2013) used a model of cue-induced conditioned activity, where discrete cues were paired with cocaine administration, to determine if the locomotor responses to cocaine sensitized during withdrawal. Rats had consistent locomotor activity following 3, 14 and 28 days of withdrawal from cocaine in this non-contingent model (Diehl et al., 2013). Conditioned place preference (CPP) models of incubation of craving have revealed variable results. Sun et al. (2017) demonstrated a decrease in CPP scores following forced abstinence from morphine whereas Li et al. (2008) found that CPP for morphine increased over a 14 day abstinence period and Lubbers et al. (2016) demonstrated an increase in CPP scores following 3 weeks of withdrawal from cocaine. In the case where CPP was not noted, animals displayed a progressive increase in side entries following abstinence from morphine suggesting enhanced drug seeking behavior (Sun et al., 2017). The use of self-administered or experimenter administered procedures may be an important factor in observing the occurrence of an incubation of craving effect. These procedural variations may also differentially engage brain regions involved in reward.

The nucleus accumbens (NAc) has been implicated in the rewarding aspects of a variety of substances and compulsive behaviors related to obtaining reward (Di Chiara, 2002; Adinoff, 2004; Chaudhri et al., 2010; Grimm et al., 2011; Nestler, 2012; Wolf and Tseng, 2012; Quintero, 2013). Specifically, the NAcSh has been shown to play a role in incentive arousal for addictive substances (Di Chiara et al., 2004). Self-administration and non-contingent administration of drug rewards has been associated with changes in immediate early genes (Renthal et al., 2008; Larson et al., 2010; Li et al., 2015a) and dendritic spine density (Christian et al., 2017) in the NAc core (NAcC) and shell (NAcSh). Cocaine that is self-administered (lever pressing) is associated with increased c-Fos labeling in the NAcSh but not in the NAcC (Larson et al., 2010). Experimenter administered amphetamine is also associated with increased c-Fos labeling but labeling decreased with repeated administration (Renthal et al., 2008). Chronic cocaine, both self-administered and experimenter administered, is associated with increased DeltaFosB labeling in the NAcSh (Larson et al., 2010). In the NAcC, both acute and chronic experimenter administered cocaine is associated with an increase in DeltaFosB (Larson et al., 2010). Chronic self-administered or experimenter administered amphetamine or cocaine is associated with increased spine density on medium spiny neurons (MSNs) in the NAcSh and NAcC (Robinson and Kolb, 1999, 2004). The increase in spine density persists months after amphetamine discontinuation and at least a month after cocaine discontinuation (Robinson and Kolb, 2004). Specific to the incubation of craving, rats trained to self-administer cocaine showed increased dendritic spine density in the NAcC following 36 days of withdrawal compared to 1 day (Christian et al., 2017).

Incubation of craving has been demonstrated for other rewarding stimuli such as sucrose (Grimm et al., 2005; Lu et al., 2007) and palatable foods (Krasnova et al., 2014; Darling et al., 2016) and is also is associated with changes in NAc immediate early genes and synaptic spines (Grimm et al., 2016; Dingess et al., 2017). Incubation of craving for sucrose is associated with elevated c-Fos-positive cells following 30 days of forced abstinence compared to 1 day in both the NAcC and NAcSh (Grimm et al., 2016). Self-administration of food reward has been shown to increase dendritic spine density within the NAcSh (Guegan et al., 2013; Mancino et al., 2017). Both high fat food and chow results in an increase in spines following 30 days of withdrawal (Dingess et al., 2017).

There are currently a dearth of studies exploring the incubation of craving for food reward comparing self-administration (contingent) and experimenter administration (non-contingent) and the associated NAc changes. Relapse from diets is around 80% (Brandon et al., 2007), thus understanding craving for food reward could contribute knowledge to the rising obesity epidemic. Relapse following dieting, even after long-term success, may be the result of long-term neural changes, such as those seen in drug addiction. The present study explored the incubation of craving for chocolate-flavored pellets using both operant (contingent) and classical conditioning (non-contingent) designs.

The operant (contingent) design followed an FR2 schedule of reward with delivery of a chocolate flavored-pellet coinciding with a discrete cue for 5 s. Studies within our laboratory have utilized an FR2 responding for chocolate-flavored pellets during operant acquisition. These studies resulted in robust responding that reached a peak by day 5 of acquisition (Holahan et al., 2010, 2012; Davis-MacNevin et al., 2013; Tuplin et al., 2015). The classical conditioning (non-contingent) design utilized a variable interval schedule of non-contingent chocolate delivery, which has yet to be studied with respect to the incubation of craving. The classical conditioning design paired a discrete cue with delivery of a chocolate flavored pellet. During testing, levers were introduced into the chamber and rats had the opportunity to bar press for the cue.

Markers of neuronal activation, FosB and c-Fos, were quantified in the NAcC and NAcSh. FosB was chosen as it is a common marker of neuron activity and addiction (Nestler, 2004, 2008, 2015) but has yet to be studied with respect to incubation of craving. The c-Fos protein was chosen because it represents an index of neuronal activity in the NAc during the behavioral testing procedure. Quantification of dendritic spines within the NAcSh was chosen because spines tend to change over time following learning and withdrawal and represent a form of structural plasticity.

## Materials and Methods

### Subjects

A total of 63 male Long Evans rats (250–275 grams) were obtained from Charles River, Quebec. Rats were housed individually in clear plastic cages (25 × 20 × 45 cm), given water *ad libitum* under a 12-h light/dark cycle (lights on at 8:00 am), and tested during the light phase. Previous operant studies within our laboratory have been undertaken during the light phase, which did not interfere with adequate acquisition of the task (Holahan et al., 2010; Davis-MacNevin et al., 2013; Tuplin et al., 2015). Food was restricted to 90% baseline, which was maintained until the end of the studies. Prior to behavioral testing, rats were handled for 5 min daily and given 10, 45-mg chocolate pellets to acclimate them to handling and the reward. Principles of laboratory animal care were followed and all procedures were conducted in accordance with the Canadian Council on Animal Care. The Carleton University Animal Care Committee approved the protocol.

### Operant Acquisition and Responding for Cues

A total of 35 rats underwent operant acquisition training, which occurred over 10 days. During acquisition sessions, rats were placed in the operant chambers (Coulburn Instruments, Whitehall, PA, USA; 30.5 W × 25.5 D × 30.5 H cm) for 1 h, where every second response (FR2 schedule) on the correct lever resulted in the delivery of a 45 mg chocolate pellet (BioServe, Flemington, NJ, USA). Once the correct lever had been depressed twice (FR2), the house light extinguished and panel lights above the lever changed from red to green for 5 s. This was accompanied by the pellet dispenser releasing one 45-mg chocolate pellet into the food hopper. The incorrect lever had no programmed consequences. Both correct and incorrect presses, nose pokes into the food hopper, and locomotor activity were recorded during the sessions. Nose pokes into the food hopper were assessed with a photocell beam-break detector that contained an infrared LED source and photodetector on the sides of the food hopper. Locomotor activity was assessed with a ceiling mounted activity monitor tuned to be sensitive to the infrared signals emitted by a rat. Movement units (brief pulses representing the minimum resolution of detection) were used to provide a measure of locomotor activity.

Following operant acquisition, rats were assigned to one of four abstinence groups and tested at different time delays in the absence of food reward. Rats were matched based on total correct presses during day 10 of acquisition to ensure all groups (abstinence periods) had obtained a similar level of acquisition. Rats were assigned to groups with the following periods of forced abstinence from chocolate: 24 h (*n* = 9), 7 days (*n* = 9), 14 days (*n* = 8), or 28 days (*n* = 9). Each rat was tested in the same operant chamber where operant acquisition training occurred. The testing session was 1 h and bar pressing no longer resulted in food reward but the discrete cues were present (the house light extinguished and the panel lights changed from red to green for 5 s). Correct presses, incorrect presses, nose pokes into the food hopper, and locomotor activity were recorded at 10-min intervals.

### Classical Conditioning and Responding for Cues

A total of 26 rats underwent classical conditioning over 10 days. During the conditioning sessions, rats were placed into the operant chambers (Coulburn Instruments, Whitehall, PA, USA; 30.5 W × 25.5 D × 30.5 H cm) for 1 h where one 45-mg chocolate pellet was dispensed on a variable interval 30-s schedule. The house light extinguishing and the panel lights changing from red to green for 5 s was accompanied by the presentation of the 45 mg chocolate pellet. Nose pokes into the food hopper and locomotor activity were recorded during each 1-h conditioning session.

Following conditioning, rats were assigned to one of four abstinence period groups (24 h (*n* = 6), 7 days (*n* = 6), 14 days (*n* = 6), or 28 days (*n* = 6)) and tested in a responding for cues behavioral design. Rats were matched based on total nose pokes on day 10 of conditioning to ensure all groups (abstinence periods) had obtained a similar level of conditioning. Testing occurred in the same chambers as training. Two levers were introduced into the operant chambers and a correct lever press resulted in the presentation of the conditioning cues (house light extinguishing and panel lights changing from red to green for 5 s) and an incorrect lever press had no programmed consequences. Rats were tested for 1 h and correct presses, incorrect presses, nose pokes into the food hopper, and locomotor activity were recorded at 10-min intervals.

### Controls

There was a second treatment where rats were food restricted but did not undergo behavioral training or testing (24 h: *n* = 3, 7 days: *n* = 3, 14 days: *n* = 3, 28 days: *n* = 3). These rats underwent food restriction for the same period of time as rats that underwent behavioral testing. Food restriction for the control rats continued throughout the 10 days that rats were behaviorally trained. The control rats were continually food restricted during the abstinence periods so that total food restriction was equal to the behaviorally trained rats. Control rats were euthanized at specific abstinence periods to determine if food restriction on its own affected immunohistochemical labeling or dendritic spine density.

### Immunohistochemistry

Ninety minutes following testing, rats were given an overdose of sodium pentobarbital and perfused with saline. Control rats were euthanized at the same time of day as behaviorally tested rats. One hemisphere was immersion fixed in 4% paraformaldehyde/0.01 M phosphate buffer solution (pH 7.4). The other hemisphere was placed in a Golgi Fix solution. Following 24 h the paraformaldehyde-immersed tissue was cryopreserved via immersion in a 30% sucrose/0.1 M phosphate buffer solution for a minimum of 4 days. The tissue was then sectioned on a cryostat at 60 μm and placed in a 0.1% sodium azide/0.1 M phosphate buffer solution (pH 7.4).

#### FosB and C-Fos

**Figure 1:**
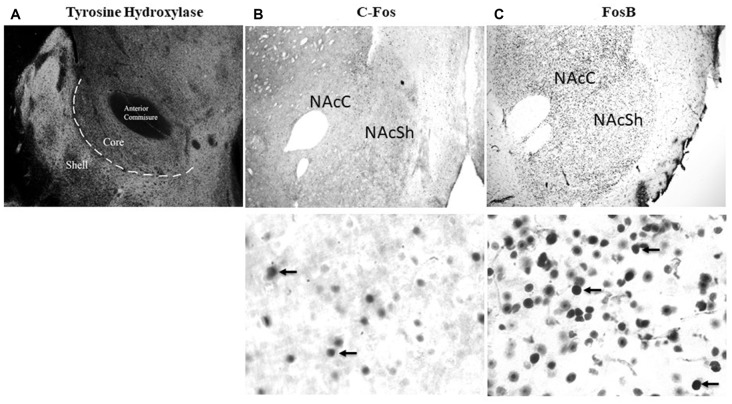
Representative images of NAc core (NAcC) and NAc shell (NAcSh) delineation and labeling. **(A)** Tyrosine hydroxylase stained image at 4x to show the location of the NAcC and NAcSh. **(B)** Representative images of c-Fos labeling in the NAcC and NAcSh at 4x and 60×. **(C)** Representative images of FosB labeling in the NAcC and NAcSh at 4x and 60×.

Two sections with the NAc from each rat were chosen for FosB and two other sections were chosen for c-Fos immunohistochemical staining. Sections were chosen from two coronal levels per rat, bregma + 2.0 mm and + 1.7 mm (Figure 1), which were averaged for analysis. This region was chosen as it has been shown to be associated with feeding behavior (Stratford and Kelley, 1997). Sections underwent three, 5-min washes in 0.1 M phosphate buffered saline with 0.1% Triton X (PBS-TX) followed by incubation in a blocking solution of 0.3% hydrogen peroxide diluted with PBS-TX for 15-min. Sections underwent three more 5-min washes in PBS-TX followed by incubation in animal free blocker (AFB; Vector Laboratories) diluted to 1× with PBS-TX from a 5× concentrated solution. Sections were incubated overnight at room temperature in the primary antibody (1:5000 anti-FosB host: mouse Cat #ab11959; Abcam or 1:5000 anti-c-Fos host: rabbit Cat #226003; Synaptic Systems). The following day, tissue underwent three 10-min washes in PBS-TX followed by incubation in the secondary antibody (FosB—1:1000 goat anti-mouse IgG: Vector; c-Fos—1:500 goat anti-rabbit IgG: Vector) for 1 h at room temperature. Next, the tissue underwent three 10-min washes in PBS followed by immersion in Avidin-Biotin Complex (ABC) for 1 h. Following ABC, the tissue underwent three, 5-min washes in PBS and was developed in 3,3’-diaminobenzidine (DAB) for approximately 5 min. Following development, the tissue was washed twice in PBS for 5 min then mounted onto slides, dehydrated and coverslipped.

#### Immunohistochemistry Quantification

A counting method based on unbiased stereology procedures using the optical fractionator method was used to estimate the number of FosB and c-Fos labeled cells in the NAcC and NAcSh subregions (see Figure 1A for delineation of these regions). The NAc was traced at 2× magnification using an Olympus BX51 bright field microscope with a motorized stage (Olympus Canada, Markham, ON, Canada) and c-Fos (examples of staining in Figure 1B) and FosB (see Figure 1C for examples of staining) labeled cells were quantified using a 60× magnification lens (oil immersion, NA 135). Counting was performed using sampling parameters sufficient to produce a Gunderson’s coefficient of error (GCE, *m* = 1) of 0.12 or less. Stereo Investigator software used planar and depth information for each counted cell to calculate the volume for the digitally traced region of interest. Counting parameters were set to a counting frame of 60 × 60 μm^2^ and a dissector height of 15 μm between the top and bottom guard zones. Estimated population number using weighted section thickness was recorded for each animal. The estimated cell count from two sections for each animal was averaged to give one datum point. Quantification is represented as an estimated total per mean measured thickness per mm^3^ to allow for comparisons across brain sections. A minimum of three animals per group were included in the analyses.

### Golgi Staining

The Golgi fixative consisted of three solutions that were prepared separately and combined in the final step of the process. Solution A required heating 750 ml of distilled water to 90°C prior to the addition of 37.5 grams (g) of mercuric chloride (Hg), the solution was then allowed to cool. Solution B required the heating of 750 ml of dH_2_O to 60°C followed by the addition of 37.5 g of potassium dichromate (K_2_Cr_2_O_7_), this solution was then allowed to cool. Solution C was made by dissolving 30 g of potassium chromate (K_2_Cr_2_O_4_) in 750 of dH_2_O. Solutions A, B, and C were then combined in a new flask and 1500 ml of room temperature dH_2_O was added. The solution was stored in a glass jar and covered in tinfoil for a least 6 days prior to use.

Following 14 days of incubation in the Golgi fixative, whole brains underwent a series of dH_2_O and sucrose washes in preparation for tissue sectioning and development. Initially, brains were immersed in dH_2_O for 4 h then immersed in fresh dH_2_O for another 3 h followed by fresh dH_2_O overnight. On the second day of washes, brains were immersed in 10% sucrose for 8 h followed by 20% sucrose overnight. On the third day, brains were immersed in 30% sucrose for a minimum of 4 days prior to sectioning on a Vibratome.

A humidified box with a plastic grate was created 24 h prior to sectioning to prevent the tissue from drying when it was slide mounted. Tissue was sectioned on a Vibratome at 200 μm and floated in a 30% sucrose solution prior to mounting. Twenty sections were taken from each brain through the NAc and placed immediately onto double dipped (2%) gelatinized slides. Slides were then placed in the humidified box in a dark cupboard for no longer than 24 h.

A desiccated box was created 24 h prior to development by placing Drierite pellets in the bottom of a plastic box that contained a plastic grate. Golgi staining was developed by immersing tissue mounted slides in several solutions, beginning with a 1-min rinse in dH_2_O. Next, slides were submerged in 28% ammonium hydroxide for 40 min and then dH_2_O for 1 min. Slides were then submerged in Kodak Film Fix A (diluted 1:1 with dH_2_O) for 40 min where the solution was kept protected from light. Next, slides were washed twice in dH_2_O prior to dehydration. For dehydration, slides were submerged in 50% ethyl alcohol (ETOH) for 1 min, 75% ETOH for 1 min, and 95% ETOH for 1 min. The remaining solutions were desiccated with molecular sieve for at least 24 h prior to use. Slides underwent three 5-min washes in 100% desiccated ETOH following by a 10-min wash in a desiccated solution of 33%ETOH, 33% Clearene and 33% chloroform. The final wash consisted of slides being placed in desiccated Clearene for 15 min. Slides were then coverslipped with Permount and placed in a desiccated box for a minimum of 3 days to allow the Permount to harden.

#### Quantification of Dendritic Spines

For each of the three behavioral groups (operant, classical, control), three MSNs within the NAcSh per rat, with a minimum of three rats per abstinence period were analyzed. This resulted in a minimum of nine neurons per abstinence period within each of the behavioral groups. The NAcSh was constricted in the anterior-posterior plane from approximately +1.7 to +3.2 mm from bregma according to Paxinos and Watson (1998). Neurons were picked at random but had to meet specific criteria to be selected. Neurons had to be entirely impregnated, staining had to be uniform and complete, the cell body had to be within the 200 μm section depth, and the neuron had to be relatively isolated from surrounding neurons. Neurons were reconstructed at 100× magnification using Neurolucida software (MicroBrightField, Williston, VT, USA). The cell body was traced first, followed by a minimum of three primary dendrites and subsequent branches, in their entirety, and all visible spines were marked. For each neuron, the total number of spines were analyzed and divided by total dendritic length to obtain the spine density of each neuron.

### Statistical Analysis

Behavioral data from each experimental design (contingent or non-contingent) were analyzed separately. Acquisition data were analyzed with a two-way, repeated measures ANOVA using abstinence period as the between factor and total correct presses (operant acquisition) or total nose pokes (classical conditioning) per day as the repeated measure. The testing data were analyzed using a two-way, repeated measures ANOVA using abstinence period as the between factor and time as the repeated measure. Correct presses, incorrect presses, nose pokes into the food hopper, and locomotor activity were analyzed separately. If ANOVAs were significant, Tukey *post hoc* comparisons were performed. Alpha was set at 0.05 for significance.

FosB and c-Fos estimated cell counts were analyzed with fixed factor ANOVAs with abstinence period and behavioral group (operant, classical, control) as independent variables. Counts from the NAcC and NAcSh were analyzed separately. Significant interactions were analyzed with one-way ANOVAs. One-way ANOVAs were also used to determine differences in abstinence periods within individual behavioral groups. Tukey’s *post hoc* analysis was used if one-way ANOVAs were significant. Alpha was set at 0.05 for significance.

Dendritic spine density analysis within the NAcSh included measurements from three neurons per rat/per brain being averaged to get one datum point per rat. Spine density was analyzed using a factorial ANOVA with abstinence period and behavioral group as the independent variables. Significant interactions were analyzed with one-way ANOVAs. One-way ANOVAs were also used to determine differences in abstinence periods within individual behavioral groups. Tukey’s *post hoc* analysis was used if one-way ANOVAs were significant. Alpha was set at 0.05 for significance.

## Results

### Operant Acquisition and Responding for Cues

**Figure 2:**
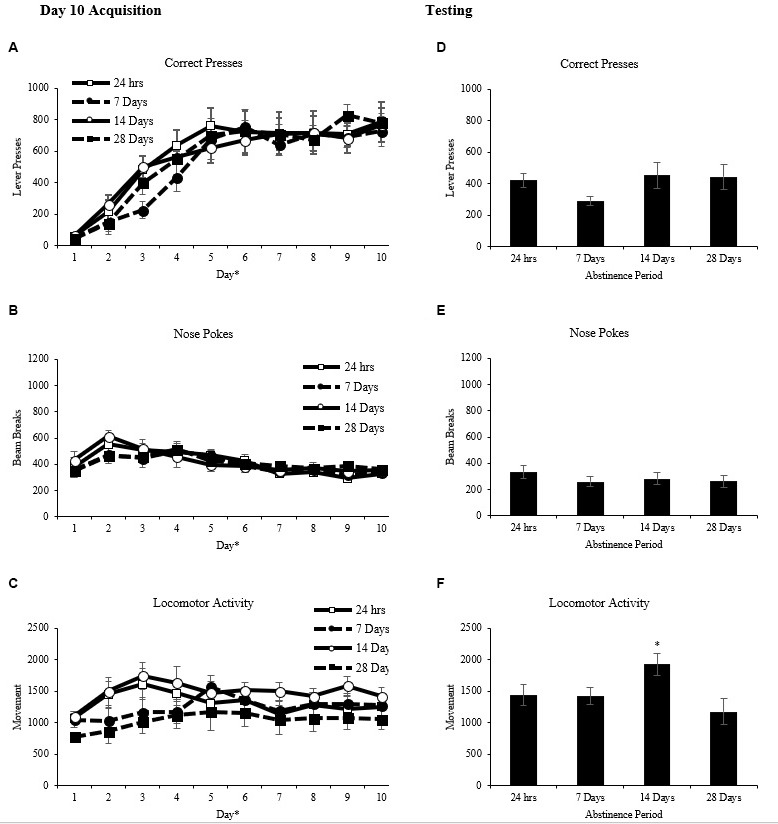
Operant conditioning (mean ± SEM). **(A–C)** Total correct lever presses, nose pokes and locomotor activity are shown for each of the 10 days of acquisition. *p < 0.01 significant effect of day. **(D–F)** Total correct lever presses, nose pokes and locomotor activity are shown for the 60-min test. *p < 0.05 compared to the 28-day abstinence period.

Rats were matched and assigned to one of four abstinence periods based on correct lever presses after day 10 of acquisition training (Figure 2A). A two-way repeated measures ANOVA on the average number of correct presses per day as the repeated measure and abstinence period as the between factor revealed a main effect of day (*F*_(9,27)_ = 62.030, *p* < 0.001))but no main effect of abstinence period (*F*_(3,31)_ = 0.265, *p* > 0.05), and no interaction (*F*_(27,279)_ = 0.769, *p* > 0.05). Nose pokes into the food hopper and locomotor activity were also assessed to ensure there were no differences between these measures. A two-way repeated measures ANOVA on the average number of nose pokes into the food hopper (Figure 2B) per day as the repeated measure and abstinence period as the between factor revealed a main effect of day (*F*_(9,27)_ = 9.327, *p* < 0.001) but no main effect of abstinence period (*F*_(3,31)_ = 0.117, *p* > 0.05), and no interaction (*F*_(27,279)_ = 0.645, *p* > 0.05). A two-way repeated measures ANOVA on the average locomotor activity (Figure 2C) per day as the repeated measure and abstinence period as the between factor revealed a main effect of day (*F*_(9,27)_ = 5.540, *p* < 0.001) but no main effect of abstinence period (*F*_(3,31)_ = 1.272, *p* > 0.05) and no interaction (*F*_(27,279)_ = 1.498, *p* > 0.05).

Rats were given one, 60 min extinction test 24 h, 7 days, 14 days, or 28 days after the last day of operant acquisition. A two-way repeated measures ANOVA on the number of correct lever presses (Figure 2D) with time (10-min intervals) as the repeated measure and abstinence period as the between factor revealed a main effect of time (*F*_(5,15)_ = 34.009, *p* < 0.01) but no main effect of abstinence period (*F*_(3,31)_ = 1.131, *p* > 0.05) and no interaction (*F*_(15,155)_ = 1.125, *p* > 0.05).

A two-way repeated measures ANOVA on the number of nose pokes into the food hopper (Figure 2E) with time (10-min intervals) as the repeated measure and abstinence period as the between factor revealed a main effect of time (*F*_(5,15)_ = 9.612, *p* < 0.001) but no main effect of abstinence period (*F*_(3,31)_ = 0.588, *p* > 0.05) and no interaction (*F*_(15,155)_ = 0.667, *p* > 0.05).

A two-way repeated measures ANOVA on locomotor activity (Figure 2F) with time (10-min intervals) as the repeated measure and abstinence period as the between factor revealed a main effect of time (*F*_(5,15)_ = 81.719, *p* < 0.001) and a main effect of abstinence period (*F*_(3,31)_ = 3.152, *p* < 0.05) but no interaction, (*F*_(15,155)_ = 1.555, *p* > 0.05). Tukey *post hoc* analysis on the main effect of abstinence period revealed that the 14-day abstinence period group had significantly higher locomotor activity than the 28-day abstinence period group, (*p* < 0.05).

### Classical Conditioning and Responding for Cues

**Figure 3:**
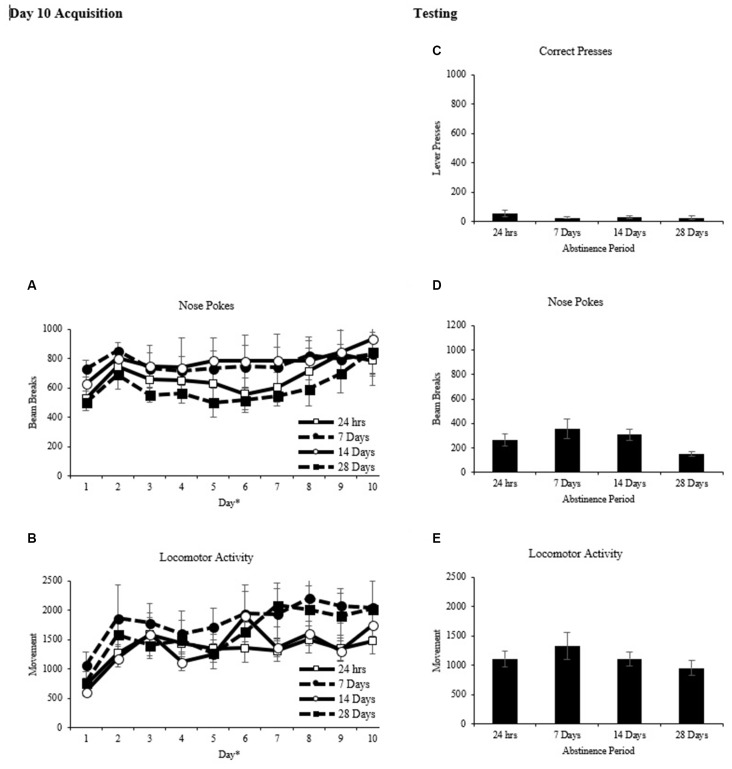
Classical conditioning (mean ± SEM). **(A,B)** Total nose pokes and locomotor activity are shown for each of the 10 days of conditioning. *p < 0.01 significant effect of day. **(C–E)** Total correct lever presses, nose pokes and locomotor activity are shown for the 60-min test.

Rats were matched and assigned to one of four abstinence periods based on nose pokes into the food hopper after day 10 of conditioning (Figure 3A). A two-way repeated measures ANOVA on the average number of nose pokes per day as the repeated measure and abstinence period as the between factor revealed a main effect of day (*F*_(9,27)_ = 4.222, *p* < 0.001) but no main effect of abstinence period (*F*_(3,21)_ = 0.716, *p* > 0.05), and no interaction (*F*_(27,189)_ = 0.386, *p* > 0.05). A two-way repeated measures ANOVA with locomotor activity per day as the repeated measure and abstinence period (Figure 3B) as the between factor revealed a main effect of day (*F*_(9,27)_ = 10.383, *p* < 0.001) but no main effect of abstinence period (*F*_(3,21)_ = 0.897, *p* > 0.05), and no interaction (*F*_(27,189)_ = 0.911, *p* > 0.05).

Rats in the classical conditioning groups were given an operant transfer test 24 h, 7 days, 14 days, or 28 days after the last day of acquisition. For the test, two levers were introduced into the chambers and pressing on one (correct) resulted in presentation of the cues that were paired with reward during training and pressing on the other (incorrect) had no programmed consequences A two-way repeated measures ANOVA on the number of correct presses (Figure 3C) with time (10-min intervals) as the repeated measure and abstinence period as the between factor revealed a main effect of time (*F*_(5,15)_ = 7.082 *p* < 0.001), but no effect of abstinence period, (*F*_(3, 20)_ = 0.818, *p* > 0.05) and no interaction (*F*_(15,100)_ = 0.880, *p* > 0.05).

A two-way repeated measures ANOVA on the number of nose pokes into the food hopper (Figure 3D) with time (10-min intervals) as the repeated measure and abstinence period as the between factor revealed a main effect of time (*F*_(5,15)_ = 11.494, *p* < 0.001) and a significant interaction, (*F*_(15,100)_ = 2.723, *p* < 0.01) but no main effect of abstinence period (*F*_(3,20)_ = 2.766, *p* > 0.05). One-way ANOVAs explored differences in nose pokes between abstinence periods at each 10-min time point (Figure 5D). A main effect of abstinence period was revealed at 20 min (*F*_(3,20)_ = 3.379, *p* < 0.05), 40 min (*F*_(3,20)_ = 3.089, *p* < 0.05) and 50 min (*F*_(3,20)_ = 5.439, *p* < 0.01) during the test. Tukey *post hoc* comparisons between abstinence periods at these time points revealed that during the 20-min interval, the 7-day abstinence period group showed significantly more nose pokes than the 28 day period group (*p* < 0.05). During the 50-min interval, the 14-day abstinence period group showed significantly more nose pokes than the 24 h and 28-day period groups (*p* < 0.05).

**Figure 4:**
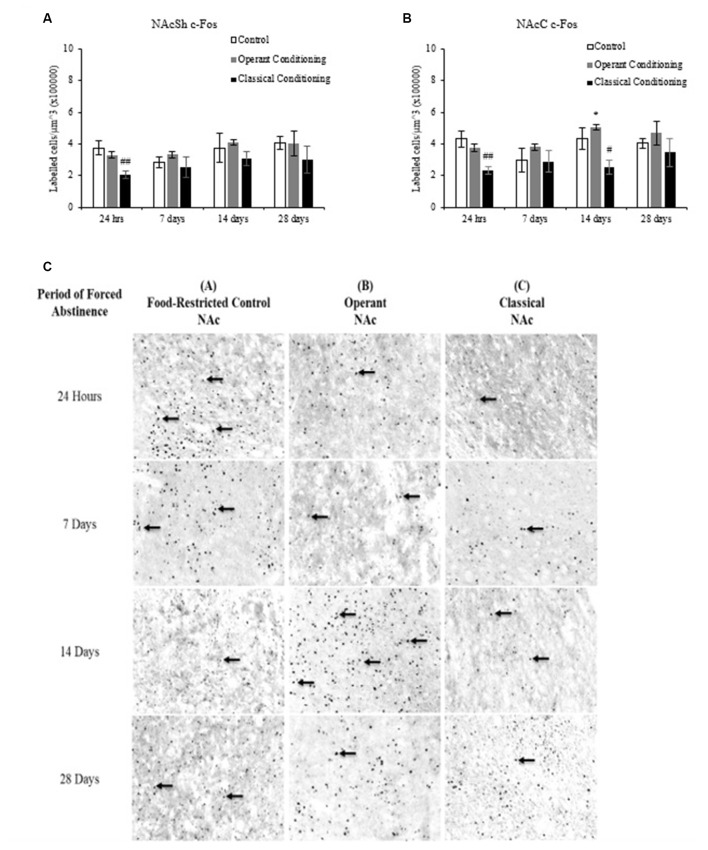
Average cell density (mean cell count/volume [mm^3^]) of c-Fos labeled neurons (mean ± SEM). **(A)** NAcSh labeled neurons in rats that underwent operant and classical conditioning compared to food restricted controls. ^##^*p* < 0.01 compared to operant conditioning and controls at 24 h. **(B)** NAcC labeled neurons in rats that underwent operant conditioning compared to food-restricted controls. *p < 0.05 compared to operant conditioning at 24 h. ^##^*p* < 0.01 compared to operant conditioning and controls at 24 h. ^#^*p* < 0.05 compared to operant conditioning at 14 days. **(C)** Representative images of c-Fos labeling in the Nucleus accumbens (NAc) in rats that underwent operant and classical conditioning and food restricted controls 20×.

**Figure 5:**
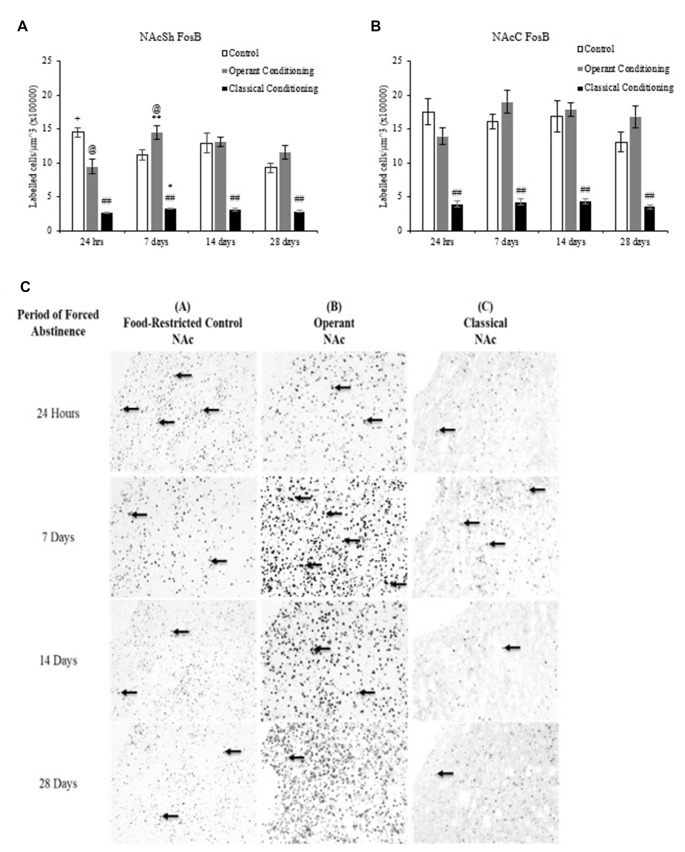
Average cell density (mean cell count/volume [mm^3^]) of FosB labeled neurons (mean ± SEM). **(A)** NAcSh labeled neurons in rats that underwent operant and classical conditioning compared to food-restricted controls. ^##^*p* < 0.01 compared to operant conditioning and control at the same abstinence period. ^@^*p* < 0.05 compared to control at the same abstinence period. *p < 0.05 compared to classical conditioning at 24 h. ^**^p < 0.01 compared to operant conditioning at 24 h. ^+^*p* < 0.05 compared to control at 28 days. **(B)** NAcC labeled neurons in rats that underwent operant and classical conditioning compared to food-restricted controls. ^##^*p* < 0.01 compared to operant and classical conditioning at the same abstinence period. **(C)** Representative images of FosB labeling in the NAc in rats that underwent operant and classical conditioning and food restricted controls 20×.

A two-way repeated measures ANOVA on locomotor activity (Figure 3E) with time (10-min interval) as the repeated measure and abstinence period as the between factor revealed a main effect of time (*F*_(5,15)_ = 38.488), *p* < 0.001 but no main effect of group, (*F*_(3,20)_ = 0.924, *p* > 0.05) and no interaction (*F*_(15,100)_ = 0.961, *p* > 0.05).

### C-Fos

A fixed factor ANOVA on the estimated density of c-Fos labeled cells in the NAcSh and NAcC (Figure 4) revealed a main effect of abstinence period in the NAcSh, (*F*_(3,31)_ = 2.920, *p* < 0.05) but not in the NAcC, (*F*_(3,31)_ = 1.735, *p* > 0.05) and no interaction between behavioral condition and abstinence period in either region (NAcSh: (*F*_(6,31)_ = 0.552, *p* > 0.05); NAcC: (*F*_(6,31)_ = 1.080, *p* > 0.05)). There was a main effect of behavioral condition in both regions (NAcSh: (*F*_(2,31)_ = 8.373, *p* < 0.01); NAcC: (*F*_(2,31)_ = 9.762), *p* < 0.01)). Tukey *post hoc* analysis revealed that in both the NAcSh and NAcC the classical conditioning group had a significantly lower c-Fos density compared to the operant and control groups (*p* < 0.05).

### FosB

A fixed factor ANOVA on the estimated density of FosB labeled cells in the NAcSh and NAcC (Figure 5) revealed a significant interaction between behavioral condition (control, operant and classical) and abstinence period in the NAcSh, (*F*_(6,36)_ = 5.472, *p* < 0.001) but no interaction in the NAcC (*F*_(6,36)_ = 1.860, *p* > 0.05). There was a main effect of behavioral condition in both regions (NAcSh: (*F*_(2,36)_ = 174.230, *p* < 0.001); NAcC: (*F*_(2,36)_ = 112.919, *p* < 0.01) but no main effect of abstinence period in either region (NAcSh: (*F*_(3,36)_ = 2.790, *p* > 0.05); NAcC: (*F*_(3,36)_ = 1.371, *p* > 0.05)). Tukey *post hoc* analyses on behavioral condition revealed that the classically conditioned group had significantly fewer labeled neurons in the NAcSh and NAcC than the operant trained and control groups at all abstinence periods (*p* < 0.01).

A one-way ANOVA on abstinence period within each behavioral condition revealed a main effect of abstinence period in the chocolate pellet treatment in the NAcSh, *F*_(3,16)_ = 5.267, *p* < 0.01. A one-way ANOVA on behavioral condition at each abstinence period revealed a main effect of treatment in the 24-h abstinence period in the NAcSh, *F*_(1,7)_ = 10.240, *p* < 0.05. A Tukey *post hoc* analysis revealed that the operant trained group had significantly more labeled neurons at 7 days than 24 h (*p* < 0.01) and that the control group showed significantly more labeled neurons at 24 h compared to 28 days (*p* < 0.05).

### Dendritic Spine Density

**Figure 6:**
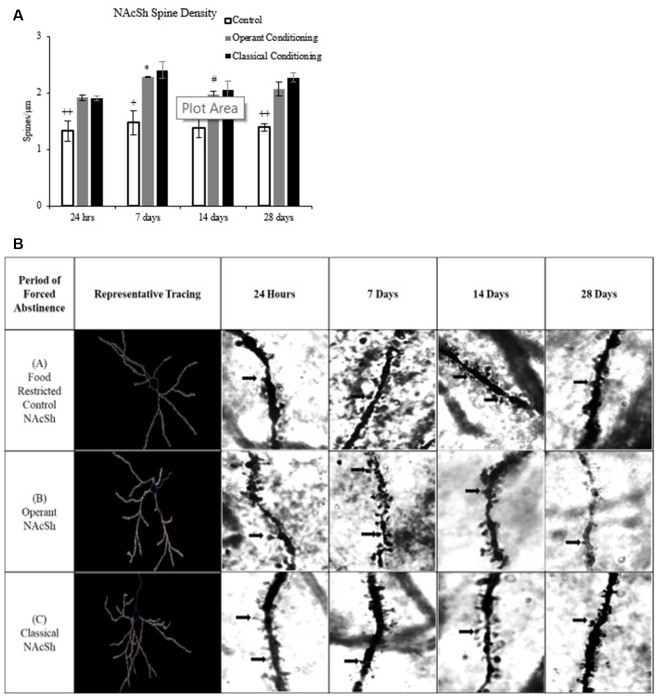
**(A)** Average dendritic spine density (spines/μm) on medium spiny neurons (MSNs) in the NAcSh in rats that underwent operant and classical conditioning compared to food-restricted controls (mean ± SEM). ^+^*p* < 0.05 compared to operant and classical conditioning at 7 days. ^++^*p* < 0.01 compared to operant and classical conditioning at same abstinence period. *p < 0.05 compared to operant conditioning at 24 h. ^#^*p* < 0.05 compared to control at 14 days. **(B)** Representative images of dendritic spines on MSNs in the NAcSh in rats that underwent operant and classical conditioning for chocolate pellets and food restricted controls 100×.

A fixed factor ANOVA on MSN spine density in the NAcSh (Figure 6) revealed a main effect of abstinence period (*F*_(3,27)_ = 3.135, *p* < 0.05) and a main effect of behavioral condition, (*F*_(2,27)_ = 39.333, *p* < 0.001) but no interaction, (*F*_(6,27)_ = 0.431, *p* > 0.05). Tukey *post hoc* analysis on behavioral condition revealed that the classical and operant behavioral conditions had a significantly higher dendritic spine density compared to the control group (*p* < 0.001).

## Discussion

### Summary of Main Findings

Incubation of craving for chocolate-flavored pellets and the impact of reinforcement contingency on craving was assessed using operant (contingent) and classical (non-contingent) conditioning behavioral designs. Following 10 days of either operant or classical conditioning using chocolate-flavored pellets as the reward, different groups of rats were tested following 24 h, 7 days, 14 days, or 28 days of forced abstinence. Behavioral measures during the test (correct lever presses, entries into the food hopper and locomotor activity) revealed similar behavioral output across all delays indicating the lack of an incubation of craving effect.

Ninety minutes following the test, brain tissue was processed and assessed for c-Fos and FosB labeling as well as dendritic spine density in the NAc. c-Fos labeling was quantified to determine neuronal activation in the NAc following each abstinence test. The pattern of c-Fos labeling revealed no incubation effect at the neural level, mirroring the behavioral outcomes. FosB was quantified to determine long-term neuronal changes in the NAc. Groups that underwent classical conditioning showed fewer FosB-labeled neurons than both the operant-trained and the food restricted control groups at all abstinence periods.

Dendritic spine density in the NAcSh was assessed to determine changes in structural plasticity. Groups that underwent either operant or classical conditioning showed significantly more dendritic spines at all abstinence periods than food-restricted controls.

### Behavioral Testing

Incubation of craving has been reported in studies examining drugs of abuse and other rewarding stimuli whereby responding for cues associated with the rewarding substance increases over extended periods of abstinence. These studies result in similar incubation effects following self-administration despite differences in training time. A time-dependent increase in responding for cocaine-associated cues has consistently been shown following extended access to self-administered cocaine (Grimm et al., 2001, 2002; Li and Frantz, 2009). In a typical model of the incubation of cocaine craving, rats are trained to self-administer cocaine for two, 3-h sessions a day for 10 days with a 40 s time-out after each reward (Grimm et al., 2002). Following each reward presentation, a 5-s tone-light cue is presented and reward is limited to 40 per 3 h session (Grimm et al., 2002). The cue-induced reinstatement test consists of a 1-h session where responses result in the presentation of a tone-light cue with a 40 s time-out (Grimm et al., 2002). A study by Li and Frantz (2009) used a similar behavioral design except training was 2 h a day for 14 days instead of 6 h a day for 10 days. Despite the differences in training, the incubation effect was similar (Grimm et al., 2001, 2002; Li and Frantz, 2009). When rats are trained to self-administer sucrose under similar conditions as cocaine, sucrose trained rats show increased responding for cues following 15 and 30 days of withdrawal compared to 24 h (Grimm et al., 2002, 2005). Rats trained to self-administer a high fat pellet for 9 h/day for 14 days emit significantly more bar presses on day 21 compared to day 2 of withdrawal (Krasnova et al., 2014). A key similarity between these reward self-administration studies is the presentation of a tone-light cue paired with the reinforcer, a time-out period following the reward, and extinction testing with cues in the absence of reward.

In the present study, neither operant nor classical conditioning resulted in an incubation of craving effect. The choice of reward, behavioral design, and rodent (mouse vs. rat) may be important factors in observing the incubation of craving phenomenon. Two notable discrepancies between our study and others is that: (1) rats were trained for 1 h. a day for 10 days; and (2) we did not utilize an extinction without cues session. Following acquisition, self-administration studies often test for reward seeking by utilizing different testing procedures which can include extinction without cues, extinction with cues, or a combination of both, thus focusing on extinction and forced abstinence models (Venniro et al., 2016). A study by Nugent et al. (2017) investigated cue-induced reinstatement of cocaine or sucrose pellet seeking in C57BL/6J mice (Nugent et al., 2017). Self-administration was 2 h/day for 3 weeks (5 days/week) and an extinction session without cues test (6 h) occurred prior to a responding for cues test. Following the extinction session, an incubation effect for cocaine, but not sucrose, was seen following 28 days of withdrawal (Nugent et al., 2017). Grimm et al. (2005) administered liquid sucrose for 6 h/day for 10 days and used testing procedures that began with extinction without cues (6 h) followed by responding for cues. An increase in responding for cues was observed following 30 days of forced abstinence compared to 1 day. The main difference between these two sucrose studies was that one used liquid sucrose/rats (Grimm et al., 2005) and the other used sucrose pellets/mice (Nugent et al., 2017). In the absence of extinction without cues, incubation of palatable food (administered 9 h/day for 14 days) and liquid sucrose (6 h/day for 10 days) craving has been demonstrated following 21 days of forced abstinence (Uejima et al., 2007; Krasnova et al., 2014).

In addition to extinction and forced abstinence models, the incubation of craving has been studied by utilizing voluntary abstinence models (Caprioli et al., 2015, 2017; Venniro et al., 2017). These studies provide a choice between psychostimulant self-administration and palatable food following periods of drug self-administration (Caprioli et al., 2017). Rats undergoing the voluntary abstinence procedure have been shown to abstain from drug self-administration when given a choice between drug and palatable food (Caprioli et al., 2017). Voluntary abstinence, Unlike forced abstinence, has been shown to prevent the development of incubation of heroin craving (Venniro et al., 2017). When the choice of palatable food is removed in rats that were trained to self-administer cocaine, cocaine seeking has been shown to increase (Quick et al., 2011). These studies provide evidence for the importance of incorporating palatable food into studies on the incubation of craving.

An additional variation in our study included the use of an FR2 schedule of reinforcement without the implementation of a time-out after reward delivery. Without the use of time-outs, it is possible rats became satiated before the end of each training period decreasing the reinforcing properties of the chocolate pellets. This is also the first incubation study that utilized chocolate-flavored pellets as the reward. Behavioral training with chocolate pellets clearly resulted in robust learning as evidenced by the increase in bar pressing over the training days and responding for the cues that continued at least 28 days following abstinence. In spite of this, there was no increase in behavioral responding over the different delay periods indicating the lack of an incubation effect. It would be worthwhile to determine if the lack of an incubation effect was due to the reward or behavioral training design by directly comparing other rewarding foods, such as sucrose or high fat pellets.

The use of non-contingent behavioral procedures in the study of incubation of craving have shown variable effects compared to contingent procedures. The CPP procedure (CPP) has been shown to elicit an incubation effect for cocaine and heroin (Li et al., 2008; Lubbers et al., 2016). In the cases where an incubation effect was not found, the preference score remained similar across abstience delays for weeks following the last administration of cocaine or morphine suggesting that the drugs were rewarding (Mueller and Stewart, 2000; Mueller et al., 2002). In another non-contingent design, conditioned locomotor responding to cocaine-associated cues was assessed (Diehl et al., 2013). Similar to the present study, cue-induced conditioned activity was observed following 3, 14, or 28 days of withdrawal without the presence of an incubation effect (Diehl et al., 2013). The studies that did not result in incubation of craving are not unlike the present study, in that responding remains consistent across abstinence periods but an incubation effect is not evident.

In the present study, rats were food restricted to 90% of baseline weight throughout the study. In other studies involving food rewards, such as sucrose and high fat foods (Grimm et al., 2005; Krasnova et al., 2014), food/water restriction was not used, or only used to initiate motivation for the reward. Food restriction may alter the rewarding effects of food and subsequent cue seeking behavior. For example, food deprivation in mice fed a high fat diet has been shown to enhance food seeking behaviors (Pérez-Ortiz et al., 2017). Food restriction has also been shown to enhance the self-administration and locomotor effects of drugs of abuse (Cabeza de Vaca and Carr, 1998). In our study, it is possible that food restriction enhanced responding for cues at all abstinence periods and prevented the observation of an incubation effect.

Food seeking behavior may also be dependent on which phase of the light/dark cycle rats are tested in. Our studies were undertaken during the light phase of the day/night cycle rather than a reversed cycle. Testing for the incubation of craving has been undertaken in the light phase (Ma et al., 2014; Nugent et al., 2017) and dark phase of the light cycle (Grimm et al., 2003; Krasnova et al., 2014) both of which have resulted in an incubation effect. While this could be a contributing factor (Roedel et al., 2006), it is likely not as important as the design of the behavioral training and testing conditions or choice of reward.

### C-Fos

Both contingent and non-contingent administration of cocaine has been shown to increase c-Fos expression within the NAcSh, but only passive administration has been shown to increase c-Fos in the NAcC (Larson et al., 2010). In the incubation of craving, c-Fos levels were elevated in the NAcC and NAcSh following 30 days forced abstinence from sucrose compared to 1 day (Grimm et al., 2016). The increase in c-Fos coincided with an increase in bar pressing following 30 days of forced abstinence compared to 1 day (Grimm et al., 2016). In the present study, c-Fos labeling remained relatively stable across all abstinence periods and was associated with the lack of behavioral incubation. This provides additional evidence that neither contingent nor non-contingent chocolate pellet delivery induces a robust incubation of craving effect.

Food-restricted controls displayed similar c-Fos labeling compared to the operant trained group. Overall, the classically conditioned group displayed lower c-Fos levels than the operant and control groups. The testing for classical conditioning involved the addition of the levers to the chambers, providing a new variable that may not be immediately associated with the reward-related cues resulting in less activation in the NAc. Cues previously associated with rewarding foods, which have acquired incentive value, have been shown to induce c-fos mRNA and c-Fos protein expression within the NAc (Figlewicz et al., 2011; Flagel et al., 2011). Besides the incentive value of food-paired cues, anticipation of scheduled food has been shown to induce c-Fos expression in the NAc (Blancas et al., 2014). Food anticipation may explain why the control group had similar c-Fos expression as the operant group. Restricted feeding schedules to normal chow has been shown to result in elevated c-Fos expression within the NAc (Mendoza et al., 2005). All animals, irrespective of group, were fed regular chow at approximately the same time every day. In behaviorally-trained groups, testing was performed approximately 1 h prior to normal feeding times and took place at similar times of day that prior training took place.

### FosB

DeltaFosB accumulates in the NAc following administration of addictive drugs and natural rewards (Kelz et al., 1999; Nestler, 2004, 2008; Larson et al., 2010; Pitchers et al., 2013; Sharma et al., 2013; Hadad and Knackstedt, 2014). The availability of extracellular DA and subsequent D1 binding may play a role in the expression of DeltaFosB within the NAc. Food restriction alone has been shown to increase DA in the NAc (Pothos et al., 1995) and increase DA receptor signaling (Carr et al., 2003). Blockade of D1 receptors in the presence of elevated DA in the NAc has been shown to attenuate DeltaFosB induction following morphine administration (Muller and Unterwald, 2005). Stamp et al. (2008) explored the effect of food restriction on DeltaFosB expression in the NAc of rats. Food restriction significantly increased DeltaFosB expression compared to free-fed controls (Stamp et al., 2008).

The response of DA release differs depending on whether a natural reward is administered in a contingent or non-contingent manner whereas DA release in response to drugs of abuse does not (Di Chiara, 2002). A study by Bassareo et al. (2015) explored the effect of response contingency on NAcSh and NAcC extracellular DA following sucrose delivery. In rats that were repeatedly fed sucrose pellets in a non-contingent manner, there was an increase in extracellular DA on the first trial that habituated on the second and third trial. In rats that underwent operant conditioning (contingent administration), extracellular DA release did not habituate over trials (Bassareo et al., 2015).

In the present study, food restricted controls displayed similar FosB expression as rats that underwent operant conditioning. This is not surprising, as food restriction and operant conditioning have been shown to increase extracellular DA. In the classically conditioned animals, the lower expression of FosB may be the result of DA habituation within the NAc in response to non-contingent administration of the chocolate flavored pellets. This could decrease DA binding to DA receptors thus resulting in less FosB expression compared to the other groups, which may have had higher extracellular DA available for binding.

### Dendritic Spines

Chronic contingent and non-contingent administration of drugs, including amphetamine and cocaine, is associated with increased spine density on MSNs in the NAcSh (Robinson and Kolb, 2004). Self-administration of food reward has also been shown to increase dendritic spine density within the NAcSh (Guegan et al., 2013; Mancino et al., 2017). In incubation of craving models, dendritic spine density has been shown to increase in the NAc following 36 days of withdrawal from cocaine and following 30 days of forced abstinence from normal chow (Christian et al., 2017; Dingess et al., 2017). These long-term changes are correlated with a variety of motivated behaviors, such as behavioral indices of craving, which may lead to relapse (Robinson and Kolb, 2004). In the present study, dendritic spine density was consistently higher at all abstinence periods in both the classical and operant groups than food restricted controls. The elevated spine densities coincide with food seeking behavior during the testing periods and may reflect long-term changes at the neural level indicative of the incentive value of reward-related cues. While spine densities were higher in both trained groups than the food-restricted controls, there were no significant differences in spine density between the operant and classical groups. These results suggest that changes in dendritic spine density may be attributed to the learning that takes place during contingent or non-contingent reinforcer delivery.

There is strong evidence that LTP associated with craving involves the insertion of new calcium permeable (CP)-AMPA receptors into the post-synaptic membrane (Malinow and Malenka, 2002). Preventing the influx of Ca^2+^ into neurons has been shown to prevent dendritic spine changes highlighting the importance of Ca^2+^ in structural plasticity (Yasumatsu et al., 2008). The amount of time necessary for CP-AMPA receptor accumulation has been shown to vary depending on the type of drug/food being administered and withdrawal time from the substance. Ingestion of “junk-food” has been shown to induce long lasting increases in CP-AMPA receptors, especially in rats susceptible to obesity (Oginsky et al., 2016). This accumulation of CP-AMPA receptors was present following 1, 14 and 21 days of junk-food deprivation, suggesting that upregulation is rapid and persistent (Oginsky et al., 2016). Following withdrawal from self-administered cocaine, CP-AMPA receptors remain similar to saline controls during the first 3 weeks followed by an increase in CP-AMPA receptors around day 30 coinciding with increased responding for cues (Wolf and Tseng, 2012). In contrast, CP-AMPA receptor accumulation following self-administration of methamphetamine is at its highest approximately 1 week after withdrawal even though seeking behavior was similar following both 7 and 30 days (Scheyer et al., 2016). In the present study, it is plausible that the expression of CP-AMPA receptors was increased in response to chocolate flavored pellets or behavioral training. The insertion of new CP-AMPA receptors could have facilitated the increase in dendritic spine density via elevated Ca^2+^ influx into the cell.

## Conclusion

Neither contingent nor non-contingent administration of chocolate flavored pellets resulted in an incubation of craving effect but resulted in robust responding across all abstinence periods. Administration of chocolate flavored pellets was associated with an increase in dendritic spine density in the NAcSh suggesting ingestion of chocolate flavored pellets synaptically alters the NAcSh for at least 28 days following forced abstinence. Differences between contingent and non-contingent administration was also evident in immediate early gene labeling. This suggests that the method of administration has differential effects on the NAc, but the functional significance of this has yet to be elucidated.

## Data Availability

The raw data supporting the conclusions of this manuscript will be made available by the authors, without undue reservation, to any qualified researcher.

## Author Contributions

ENT and MH conceived and designed the experiments and wrote the manuscript. ENT and SL performed the experiments. ENT analyzed the data.

## Conflict of Interest Statement

The authors declare that the research was conducted in the absence of any commercial or financial relationships that could be construed as a potential conflict of interest.
